# 泽布替尼治疗伴冷球蛋白血症的华氏巨球蛋白血症1例

**DOI:** 10.3760/cma.j.cn121090-20241105-00433

**Published:** 2025-05

**Authors:** 昱翀 秋, 路 张

**Affiliations:** 1 中国医学科学院，北京协和医学院，北京协和医院血液内科，北京 100730 Department of Hematology, Peking Union Medical College Hospital, Chinese Academy of Medical Sciences and Peking Union Medical College, Beijing 100730, China; 2 中国医学科学院，北京协和医学院，临床医学专业试点班，北京 100730 4+4 Medical Doctor Program, Chinese Academy of Medical Sciences and Peking Union Medical College, Beijing 100730, China

患者，女，68岁，因“遇冷后皮肤青紫6年”于2017年6月就诊于北京协和医院。患者2011年遇冷后出现皮肤青紫，未在意，未诊治。2014年外院检查提示“轻度贫血，血IgM水平升高”。2017年6月，患者遇冷后症状加重，同时出现头晕、视物模糊，伴雷诺现象和皮肤网状青斑，血常规提示中度贫血（HGB 74 g/L），标本有凝集现象。冷凝集素1∶1 280阳性，Coombs试验阳性，IgM 69.95 g/L（血免疫固定电泳提示IgM κ），冷球蛋白阴性，眼底检查提示双眼视网膜出血。骨髓涂片、骨髓流式细胞术、骨髓活检均支持华氏巨球蛋白血症（WM）。进一步基因检测提示MYD88 L265P突变阳性，CXCR4野生型。查体示贫血貌，浅表淋巴结不大，肝脾肋缘下未触及。因存在冷凝集素病及症状性高黏滞综合征，有治疗指征，首疗程治疗予氟达拉滨+环磷酰胺（FC）方案，第2个疗程应用利妥昔单抗联合FC方案。因骨髓抑制严重，第3个疗程化疗采用利妥昔单抗+环磷酰胺+地塞米松方案。由于用药后骨髓抑制仍严重，且已达到非常好的部分缓解，3个疗程后暂停化疗。化疗后患者HGB持续正常，IgM最低降至4.14 g/L。2022年4月（距末次化疗已近5年），患者再次出现IgM水平升高（由4.14 g/L升至15.46 g/L），但无贫血，无明显症状，继续观察。2022年10月，患者再次出现遇冷后皮肤青紫，伴乏力、食欲下降，仍无贫血，IgM水平升高至26.65 g/L。冷球蛋白检测提示Ⅰ型冷球蛋白血症，冷球蛋白定量为1 457.1 mg/L。考虑WM进展合并Ⅰ型冷球蛋白血症，存在治疗指征。因既往化疗骨髓抑制重，患者2022年10月开始接受泽布替尼单药治疗（160 mg每日2次口服）至今。用药后患者乏力及遇冷皮肤青紫症状改善，食欲恢复，IgM降至6.5 g/L，冷球蛋白降至617.8 mg/L，疗效评估达到部分缓解。患者现接受泽布替尼治疗已超过2年，无明显不良反应，耐受良好。

讨论：WM是一种少见的惰性成熟B细胞淋巴瘤，部分患者可合并冷凝集素病和（或）冷球蛋白血症。冷凝集素病是一种自身免疫性溶血性贫血，遇冷条件下，IgM型冷凝集素导致红细胞凝集并激活补体通路，最终引起溶血并导致贫血。冷球蛋白血症虽然也会有遇冷相关表现，但核心为遇冷条件下出现沉淀的冷球蛋白（本例患者为IgM κ）。本例WM患者在病程中先后出现冷凝集素病和冷球蛋白血症，提示肿瘤产生的IgM在疾病不同阶段具有不同的生物学行为。既往类似病例多采用以利妥昔单抗为基础的化疗方案，虽可取得较好疗效，但可发生包括骨髓抑制在内的严重不良反应。本例患者既往对此类治疗耐受性较差，故疾病进展后更换了治疗策略。对于存在MYD88突变且CXCR4为野生型的WM患者，布鲁顿酪氨酸激酶抑制剂疗效较好。本例患者采用泽布替尼治疗取得了较好疗效，虽然疗效仅达到部分缓解，但考虑到症状缓解而非缓解深度是治疗WM的首要目标，对于治疗有效的患者，不宜频繁更换治疗方案。另外，由于患者的冷球蛋白血症继发于WM，故采用泽布替尼治疗原发病对控制冷球蛋白血症也有一定的作用。本例是首个在冷球蛋白血症患者中应用泽布替尼治疗的成功病例，初步提示了泽布替尼对冷球蛋白血症患者的潜在应用价值。

